# Supplementary material for: Overexpression of MicroRNA-138 Affects the Proliferation and Invasion of Urothelial Carcinoma Cells by Suppressing SOX9 Expression
Source: Biomedicines. 2023 Nov 15;11(11):3064. doi: 10.3390/biomedicines11113064 (PMC10669193; doi:10.3390/biomedicines11113064)

Figure S1

SOX9 mRNA expression after transfection with various microRNA (miRNA) precursors

Relative expression of SOX9 mRNA after transfection of T24 and UMUC2 urothelial carcinoma cell lines with 11 miRNA precursors.

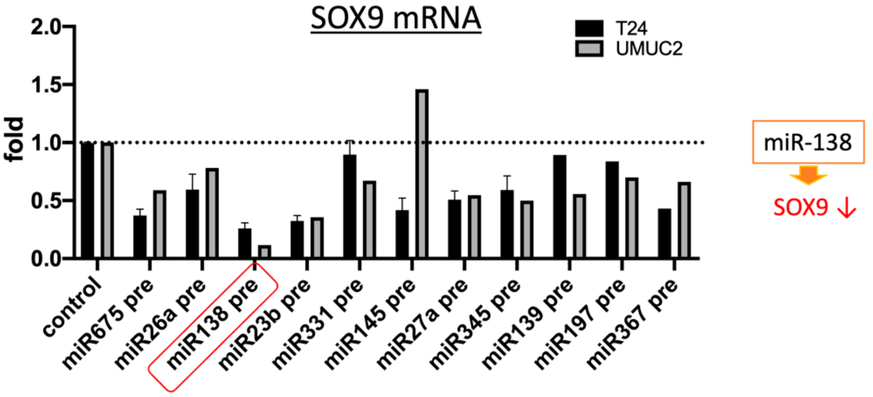

Figure S2

Putative binding site of miR-138 on SOX9 mRNA.

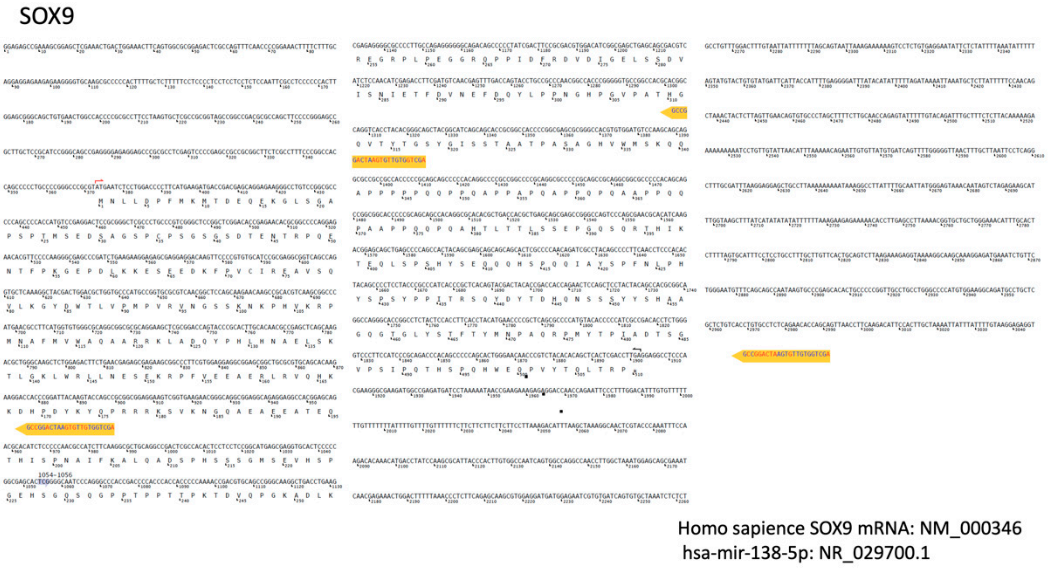

Supplement: Supplementary file 1 [file biomedicines-11-03064-s001.zip › biomedicines-2692115-supplementary.pdf]
